# Immunological Dysregulation in Multiple Myeloma Microenvironment

**DOI:** 10.1155/2014/198539

**Published:** 2014-06-11

**Authors:** Alessandra Romano, Concetta Conticello, Maide Cavalli, Calogero Vetro, Alessia La Fauci, Nunziatina Laura Parrinello, Francesco Di Raimondo

**Affiliations:** ^1^Department of Clinical and Molecular Biomedicine, Haematology Section, University of Catania, Catania, Italy; ^2^Fondazione Veronesi, Roma, Italy; ^3^Division of Hematology, AOU “Policlinico-Vittorio Emanuele”, Catania, Italy

## Abstract

Multiple Myeloma (MM) is a systemic hematologic disease due to uncontrolled proliferation of monoclonal plasma cells (PC) in bone marrow (BM). Emerging in other solid and liquid cancers, the host immune system and the microenvironment have a pivotal role for PC growth, proliferation, survival, migration, and resistance to drugs and are responsible for some clinical manifestations of MM. In MM, microenvironment is represented by the cellular component of a normal bone marrow together with extracellular matrix proteins, adhesion molecules, cytokines, and growth factors produced by both stromal cells and PC themselves. All these components are able to protect PC from cytotoxic effect of chemo- and radiotherapy. This review is focused on the role of immunome to sustain MM progression, the emerging role of myeloid derived suppressor cells, and their potential clinical implications as novel therapeutic target.

## 1. Introduction

Multiple myeloma (MM) is a systemic hematologic disease due to uncontrolled proliferation of monoclonal plasma cells (PC) in bone marrow (BM). MM symptoms depend on organ damage and include renal failure, anemia due to extensive BM infiltration, hypercalcemia, and pain due to osteolytic bone lesions [1]. A monoclonal proliferation of PC is also present in other conditions that can be considered as preclinical phases of MM, including monoclonal gammopathy of undetermined significance (MGUS) and asymptomatic or smoldering myeloma (SMM). Recent studies have documented that virtually all cases of MM pass through a MGUS phase, although it is often not recognized [2, 3]. The rate of evolution from these preclinical conditions to an overt myeloma is very low and it has been calculated to be 1% per year for MGUS and 10% per year for SMM.

However, while both MGUS and SMM lack the clinical features of MM, they harbor the same genetic alterations of symptomatic myeloma [4, 5]. Therefore, the transformation of MGUS to MM seems to be a multistep process where several factors may play a role. Besides additional, acquired genetic and epigenetic changes of PC, it is likely that a “permissive” microenvironment plays a significant role in the evolution from MGUS to symptomatic myeloma [6].

In fact, a well-recognized feature of MM is the presence of an intimate relationship between PC and bone marrow microenvironment where PC are hosted in special niches and receive multiple signals that maintain their long survival and exert a protective effect on drug-induced apoptosis. Therefore, as emerging in other solid and liquid cancers, the host immune system and the microenvironment have a pivotal role for the PC growth, proliferation, survival, migration, and resistance to drugs and are responsible for some clinical manifestations of MM.

In addition to the cellular component of the microenvironment (represented by stromal cells, osteoblasts, osteoclasts, adipocytes, endothelial cells, and T and natural killer cells), PC interact with extracellular matrix (such as laminin and fibronectin), adhesion molecules (including syndecan1, VCAM1, and VLA4), cytokines (the most studied being IL-6, TNF*α*, HGF, and IGF), and growth factors, produced by both stromal cells and PC themselves. All these components are able to protect PC from cytotoxic effect of chemo- and radiotherapy [7, 8]. Indeed, MM PC are not able to grow in a medium without a stromal support and even PC from patients affected by refractory myeloma show drug sensitivity when cultured in vitro [9].

This review is focused on the role of microenvironment and immunome to sustain MM progression and resistance to chemotherapy.

## 2. MM Microenvironment: In Vivo Models

MM microenvironment has been evaluated in vitro and in several murine models [10], all of them limited for reproducible, convenient, and sensitive monitoring of cellular immunological changes [11]. Currently available murine models for MM include immunocompetent mice, such as the 5TMM series [12, 13] and genetic models of MM [14, 15], or immunocompromised mice, namely, NOD/SCID [16], SCID-hu [17, 18], and NOG [19, 20].

The 5TMM and the genetic models of MM have the advantage of affording preclinical studies in immunocompetent hosts, but molecular and biological differences exist between murine and human MM cells [10]. The number of available murine genetic models of MM and of 5TMM cell lines is extremely restricted and does not represent the heterogeneity of the human disease. Immunological changes can be monitored but are limited to the mouse background and cannot be translated* tout-court* in MM patients.

Moreover, MM is different when developed in mouse or human background [21]. Thus, an emerging need is the development of murine models with both cancer and microenvironment in human background. Available xenograft models of human myeloma into mice to study MM expansion in immune human system include NOD/SCID mice, SCID-Hu model using human fetal bone [21], and SCID-Rab using rabbit bones implanted subcutaneously in unconditioned SCID mice [22].

SCID models have been further modified using synthetic scaffolds instead of fetal human bone to study the expansion of human primary MM cells [23] or using a silicomodel that allows visualizing in a three-dimensional space the dynamics of BM microenvironment and the relevant role of SDF1/CXCR4 axis [24].

Immunodeficient RAG2−/−*γ*c−/− mice (that completely lack B, T, and NK cells) have been used to investigate the graft versus myeloma effect and T-cells modulation from human peripheral blood mononucleated cells PBMC, showing that human PBMC can be safely inoculated in mice to investigate microenvironment. Human mesenchymal stromal cells and bone particles can be also implanted in mice to create a humanized environment for MM cells and bioluminescent imaging is used to follow in a noninvasive way engraft, growth dynamics, and response to therapy of patients PC [25].

However, in these models, immunological impairment present in MM patients is still hard to monitor since the whole immunome cannot be modelled, due to excess of inflammation in models using synthetic scaffolds or the presence fetal immunome in those using fetal bone scaffolds.

However, thanks to these models we addressed many details about the interaction of PC in BM, thus to define the MM “niche” and their relative likely or known relation to myeloma genesis.

## 3. The MM Niche

The main components of MM niche are [26, 27]

1. extracellular matrix (ECM): fibrous proteins, proteoglycans, glycosaminoglycans, and small integrin-binding ligand N-linked glycoproteins,
2. soluble component: cytokines, growth factors, and adhesion molecules,
3. hematopoietic and nonhematopoietic cells: bone structural cells (stromal cells, adipocytes, osteoclasts, and osteoblasts), immune cells (T lymphocytes, dendritic cells), and the vasculature.

ECM is the dynamic substrate that supports cells anchorage to BM niche and regulates growth factors distribution. Fibrous proteins (such as collagen, laminin, fibronectin, and elastin), proteoglycans (such as heparan sulfate-containing proteoglycans and small leucine-rich repeat proteoglycans (SLRPs): decorin, biglycan, fibromodulin, and lumican), glycosaminoglycans (particularly hyaluronan), and SIBLING proteins (such as osteopontin, bone sialoprotein (BSP), and dentin matrix protein-1 (DMP-1)) constitute ECM. In MM patients ECM composition is altered and variably disorganized [28].

Autocrine and paracrine loops and cell-cell adhesion mechanisms regulate PC production of cytokines and growth factors within the BM microenvironment. These different components are able to induce signaling pathways responsible for PC survival, growth, and migration among which the most important are Ras/Raf/MEK/MAPK pathway, PI3 K/Akt pathway, the JAK/Stat3 pathway, the NF*κ*B pathway, and the wingless-type (Wnt) pathway [29–34].

It has been widely described that NF-*κ*B transcription factor plays a key role in the pathogenesis of multiple myeloma within BM microenvironment where there is an increased MM expression and activation of molecules involved in both the canonical and noncanonical NF-*κ*B pathway [29, 35]. NF-*κ*B signaling pathways play an important role not only for MM cells but also for many other types of stromal cells by inducing the production of prosurvival cytokines such as IL-6, BAFF, or APRIL [36].

Our group has recently described that the majority of MM cells from BM specimens at different stages of disease almost exclusively express the cytoplasmic (inactive) form of NF-*κ*B while, in mesenchymal cells from MM-patients, NF-*κ*B is present in the nuclear active form, further underlining the relevance of BM mesenchymal cells [37]. In addition, the proteasome inhibitor bortezomib, which was described in the past as a NF-*κ*B antagonist, had a consistent antitumor activity against both chemoresistant and chemosensitive MM cells, regardless of the NF-*κ*B localization, thus suggesting the existence of other molecular targets of proteasome inhibitors in MM [29, 35, 37]. The overexpression and activation of several molecules involved in NF-*κ*B signaling give rise to promising targets for novel anti-MM therapy [38].

Two types of cells primarily compose the cellular compartment of the BM niche: hematopoietic and nonhematopoietic cells, including the vasculature. Among the nonhematopoietic cells (stromal cells, including pericytes, marrow adipocytes, fibroblasts, osteoblasts, osteoclasts, and endothelial cells), we will focus on osteoblast/osteoclast ratio and on the vasculature.

Bone homeostasis is normally maintained by the opposite activity of osteoblasts and osteoclasts. BM malignant PC induce osteoclastogenesis and inhibit bone building by osteoblasts, thus altering bone homeostasis and leading to bone damage. The general view is that MM PC adhere to BM stroma and induce the secretion of different proosteoclasts and antiosteoblasts cytokines. The adhesion is mediated by integrins such as CTLA4-1 integrin and VLA-4 expressed by MM cells and VCAM-1 expressed by stromal cells. This interaction induces osteoclasts resorbing activity and osteolysis [39]. After adhesion, increased bone resorbing activity by osteoclasts and MM survival is mediated by a variety of osteoclast-activating factors, such as macrophage inflammatory protein-1*α* (MIP-1*α*), receptor of NF-*κ*B ligand (RANKL), VEGF, TNF-*α*, IL-1*β*, HGF, and IL-6, produced by both tumor as well as stromal cells. In particular, RANKL is a member of TNF family whose antagonist is osteoprotegerin (OPG) ligand. When MM cells adhere to stromal cells, they induced expression of RANKL by BM stromal cells, thus promoting osteoclast activity and differentiation. Conversely, decreased number and bone formation activity of osteoblasts are associated with dysregulation of several signaling molecules, among which are dickkoppf 1 (DKK1), IL-3, and IL-7. DKK1 is overexpressed in MM patients with bone lesions. It is able to inhibit Wnt signaling pathway, critical for osteoblast differentiation, and to abolish Wnt-related OPG production. The cytokines IL-3 and IL-7 negatively affect osteoblast survival. Importantly, the main source of IL-3 is the BM CD3+T lymphocytes, suggesting an additional role for T lymphocytes, which also overproduce RANKL in MM BM with bone damage [39, 40]. This complex network of cytokines and signaling pathway induce osteoclasts resorbing activity and osteolysis.

As for bone homeostasis, the balance between proangiogenic and antiangiogenic factors is lost in MM BM niche in favor of neoangiogenesis. Angiogenesis (evaluated in MM BM specimens by microvessel density, MVD) is increased in patients with active myeloma in comparison with MGUS or smoldering MM patients. An “angiogenic switch” is a feature of active myeloma, as a “vascular phase” of disease, while MGUS and smoldering myeloma are arrested in an “avascular phase” [41, 42]. Increased MVD is a poor prognostic factor at diagnosis for patients who undergo high dose chemotherapy with autologous transplant [43].

Angiogenesis in MM is the result of physical factors such as hypoxia and chemical substances such as hypoxia-inducing factors (HIFs), VEGF, fibroblast growth factor (FGF), hepatocyte growth factor (HGF), angiopoietin, platelet derived growth factors (PDGF), and endothelial growth factor (EGF), whose concentration is much higher in BM than in peripheral blood of MM patients [44]. In particular, microenvironment VEGF, produced by both MM PC and BM stromal cells, is able to induce MM growth and survival and egress from BM via its VEGFR-1 receptor on MM cells and to induce angiogenesis through VEGFR-2 on endothelial cells.

In many MM mice models, hypoxia is a specific feature of MM microenvironment. Moreover, the pathways of HIFs, VEGFs, and VEGF receptors are upregulated in the majority of MM cases and are associated with angiogenesis. Both VEGF and HIF could be a therapeutic target of anti-MM therapy with specific small-inhibitor molecules even if many common anti-MM drugs such as bortezomib and lenalidomide are able to inhibit HIF-1*α* activity and thalidomide-derived immunomodulatory drugs (IMiDs) including lenalidomide and pomalidomide which are* per se* antiangiogenetic drugs [45].

## 4. Immunome: An Emerging Role in MM Microenvironment

Among the hematopoietic cells, we can consider a variety of cells from hematopoietic stem cells (HSCs) and mesenchymal cells (MSC), to mature erythrocytes, megakaryocytes, platelets, immune cells, such as B and T lymphocytes, natural killer (NK) cells, macrophages, and dendritic cells (DCs).

Macrophages support survival and stimulate proliferation of MM cell lines in vitro and protect PC cells from spontaneous and drug-induced apoptosis, thanks to secretion of IL-6 and vascular endothelial growth factors [46]. Eosinophils contribute to MM cells proliferation in a largely contact-independent manner, though not by IL-6 or APRIL, usually produced by many other kinds of BM cell types to support normal and malignant PC survival and proliferation [47].

An impairment in the function of immune cells has been widely described in MM patients [48]. MM patients have a greater susceptibility to infection and secondary malignancies and this is the clinical counterpart of an intricate cellular interaction involving the PC clone and the BM microenvironment [49]. The MM-related immunological dysfunction is also able to model PC activity and damage capacity [49]. Indeed, the PC-induced modulation of the surrounding microenvironment involves also host immune effectors.

The levels of B cells, NK cells, and CD4+ T cells are inferior when compared to normal control as well as the immunoglobulin reduction which indicate a typical clinical feature of symptomatic MM [49].

The main dysregulated immunological elements include: Treg, TH17, MDSC, and DC. However, the regulation of the immune-effector cells is the result of a complicated cross talk between PC, DC, and CD4+ cells, through indoleamine 2,3 dioxygenase, the programmed death 1-ligand, and the B7H3 action [50, 51].

### 4.1. Dendritic Cells

The functions of the antigen presenting cells DCs are defective. The direct interaction between DCs and PC resulted in PC survival and spontaneous cell-cell fusion with formation of giant bone resorbing cells. In a recent model of DCs-MM and T lymphocytes interactions in BM microenvironment, the expression of the costimulatory molecule CD28 on MM plasma cells and of ligands CD80/CD86 (that normally activate T cells) on BM DCs induced DCs to produce both IL-6 and the immunosuppressive enzyme indoleamine 2,3 dioxygenase (IDO). IL-6 is one of the most important MM prosurvival factors while IDO is able to deplete an essential amino acid whose absence induces anergy of activated T cells and differentiation of T cells in suppressive CD25^high^/FOXP3^+^/CD4^+^ Treg cells. Blockage of CD28 inhibits IL-6 and IDO production by DCs thus abrogating the protective effect of DCs on MM cells [52].

### 4.2. Treg

Development of CD4 T cells is a plastic process influenced by cytokines of the microenvironment milieu. A paradigm of the adaptive immunity describes in detail the balance between Th1/Th2 CD4 subpopulations, due to specific soluble factors.

Th1/Th2 balance in MM is altered with a reduced production of Th1-like cytokines, such as IL-2 or IFN-*γ*, with an overexpression of Th2 cytokines, IL-10 and IL-4 [53]. Similarly, a plastic balance between Th1, Treg, and Th17 is emerging, driven by TGF-*β*. The forkhead/winged helix transcription factor forkhead box P3 (Foxp3) expression identifies that Treg and retinoid-related orphan receptor (ROR-*γ*) are a marker of Th17. Naïve T cells overexpress ROR-*γ* and Foxp3, and in presence of TGF-*β* Foxp3 inhibits ROR-*γ* leading to Treg expansion. However, in presence of proinflammatory stimuli, including IL-6, IL17-*α*, and IL17-*β* this inhibition does not occur thus switching to Th17 phenotype [54–59], promoting Th17 expansion, and inhibiting Treg differentiation.

MM-derived Tregs are unable to regulate T-cell expansion and function [60, 61]. Tregs behavior remains a critical debate in MM [62], due their source (peripheral blood versus bone marrow), the quantification method (absolute count versus percentage), and the immunophenotype used. Some studies showed increased CD4^+^CD25^high^Foxp3^+^ Treg with inhibitory functions [63, 64], while others, including data from our group (Parrinello, manuscript in preparation) show lower absolute numbers in MM patients compared with healthy volunteers [65, 66]. In some series MM Tregs were dysfunctional, because of being unable to inhibit anti-CD3 mediated T-cell proliferation [66]. However, FOXP3 expression is not a definitive and unique marker for Tregs, these cells being also characterized as CD3^+^CD4^+^CD25^+^CD127^low^ cells [67, 68].

### 4.3. Th17

Th17 produce IL-17 and IL-22 cytokines and, as above described, they are strongly related to the effect of IL-21, IL-22, IL-23, and IL-27, whose levels are augmented in MM [69]. PC express the IL-17R (receptor of IL-17) on the cell surface, thus being sensitive to the survival stimuli given by IL-17 cells, as shown in vitro, in murine model and in patients [69]. Several studies indicate an increase of Th17 in the PB and BM of MM patients [69–72], as consequence of increased proinflammatory cytokines leading to Th17 polarization in MM milieu. Th17 expansion along with increased IL-17 suppress immune responses, protect PC from CTL attack, and promote their survival and growth.

Additionally, IL-17 plays a pivotal role in the development of bone disease, since the higher the levels of IL-17 are, the more advanced the bone disease is. IL-17 is also able to determine an upregulation of RANKL on stromal cells thus determining a stimulation of osteoclasts and so the generation of bone lesions [70]. The amount of Th17 in BM is positively correlated to lytic lesions [70], clinical tumor stage, serum lactate dehydrogenase concentration, and serum creatinine concentration [72].

Th17 are a potential predictive factor of therapy response [72]. Curiously, the enrichment of BM with Th1 cells is able to revert this phenomenon [70]. However, a multivariate analysis of these factors is missing.

The relationship between Th17 and Th1 cells [57] could depend on the microenvironment signature, in particular on the function of DC, through IL-6 secretion [73]. A balance between Th17/ and Treg in favor of Th17 has been observed in long-term MM survivors compared to short-term MM survivors [71]. However, the real Treg/Th17 interaction has not completely been defined since some reports indicate that these constitute a cell subset essential for disease progression [69, 70], while other reports indicate that these cells could have an antitumor activity, regulating cell-to-cell cross talk and leading to a long-term control of the disease [71].

It is likely that the study of the whole microenvironment, taking together the myeloid and lymphoid axis, would be useful in the definition of disease pathogenesis and its prognostic implication.

An indirect marker of T-cell dysfunction could be the evaluation of CD200+T cells. CD200 is a transmembrane glycoprotein belonging to the immunoglobulin superfamily, physiologically expressed on lymphocytes to induce immune tolerance, for example, during pregnancy to avoid conflicts between mother and newborn's immune system. Since CD200 is expressed by different tumor cell types [74–77], it has recently described the relevance of the CD200-CD200R axis in cancer immune evasion. In fact, the expression of the cognate ligand of CD200, CD200R, is restricted to certain populations of T cells and mainly to myeloid-derived Ag presenting cells, among which tumor-associated myeloid cells whose suppression, that is, in melanoma model, is able to abrogate tumor formation [78]. The first data on a likely anti-CD200 treatment in neoplastic diseases come from hematologic malignancies where it has been demonstrated that CD200 blockade may represent a novel approach to clinical treatment of CLL [79].

Increased expression of immune-regulatory molecule CD200 has been described also in MM where it is accepted as a negative prognostic factor [80, 81], but it has never been investigated on T-cell subsets in MM.

Our group has recently confirmed that almost the 70% of MM cells in BM specimens express CD200 and, as in melanoma, the dependence of CD200 expression on RAS/RAF/MEK/ERK pathway. Furthermore, on the basis of a reduced immunogenicity in vitro of CD200-positive cells depending on ERK pathway, explored by mixed lymphocytes cultures, we have supposed that CD200 expression in MM could suppress antitumor response in bone marrow microenvironment suggesting that an anti-CD200 treatment could be therapeutic in MM [82].

Our data do not allow speculating any role for myeloid involvement in this pathway, although CD200R is an inhibitory immune receptor initially described mainly on myeloid cells [83].

### 4.4. Myeloid Derived Suppressor Cells

Accumulation of myeloid derived suppressor cells (MDSC) has been described in the peripheral blood of patients affected by solid tumors [84–86], and only a few reports are focused on MM, including 11 newly diagnosed patients [87], 13 relapsed/refractory patients [88], and 11 MGUS [89–91].

MDSC favor the tumor escape from immune-surveillance. MDSC exert an immunosuppressive activity mainly on T lymphocytes because of high levels of arginase, which is able to deplete the microenvironment of arginine, an essential amino acid for T-cell activity. MDSC induce also inhibition of T-cell receptor, by nitrosylation, and ROS release and in this way create conditions for cancer progression as well [92].

In tumour-bearing mice, MDSC can be identified in tumor infiltration and in the spleen as myeloid cells at various maturation stage CD11b+Gr1+ and based on the expression of Ly6G can be distinguished in granulocytic and monocytic fractions.

In humans, several MDSC subpopulations have described termed monocytic (mo-MDSC, CD14^+^HLA-DR^low/−^) and granulocytic MDSC (G-MDSC, CD33^+^CD14^+^HLA-DR^low/−^), respectively, in absence of a human marker equivalent to Ly6C in mice.

The role of MDSC in MM progression is currently under investigation. Most of our current knowledge on MDSC is obtained by studying solid tumors that expand MDSC in the lymphoid organs, but information on whether cancer cells residing in the bone marrow (BM) are able to directly influence MDSC generation in situ in MM has been recently evaluated by three groups [87, 88, 93].

Using a 5T-2MM murine model [93], in which the MM cells grow in the BM in fully immunocompetent mice, no dramatic changes in the relative abundance of MDSC subsets were noted, suggesting that MDSC expansion is an early event in MM. The same happens in MM patient's bone marrow, where MDSC infiltration is hard to define because of neoplastic plasma cell expansion. However, when the amount of MDSC was calculated as percentage of the nonneoplastic cells (identified as CD138 negative cells), MDSC accumulation in BM of MM patients was evident when compared with BM healthy donors (41.1 (range, 13.3–75.9%) versus 22.9 (range, 7.7–33.3%)) [87]. Data from Dr. Gorgun confirmed an increase of MDSC in the bone marrow, but it was compared to peripheral blood and not bone marrow in healthy subjects [88].

In the murine model, T-cell suppressive capacity of 5T2MM MDSC subsets could already be observed after 3 weeks—a time point at which the tumor load is very low—and was maintained throughout weeks 6, 9, and 12, confirming that MDSC immunosuppression is an early event in MM disease [93]. Using another immunocompetent mouse model, established by intravenous inoculation of BCM, DP42, or ATLN MM cells into syngeneic mice, MDSC accumulated in BM as early as 1 week after tumor inoculation. When these mice were engineered to lose their ability to accumulate MDSC in tumor-bearing hosts (S100A9 knockout), growth of the immunogenic MM cells was significantly reduced showing again that the accumulation of MDSC at early stages of MM plays a critical role in MM progression [87]. In the ATLN model, a significant increase in the proportion and absolute number of MDSC in BM was observed as early as 1 week after tumor cell inoculation, followed in weeks 2-3 by a reduction due to MM expansion in BM and a progressive increase in spleen and lymphonodes. MDSC continued to grow during week two posttumor injection, reflecting the fact that MM cells accumulated in spleen at later time points and to a lesser extent than in BM. In fact, only at the end of week 3 (late MM stage in mouse model), the presence of MDSC in spleens declined, without any difference in the kinetics for G-MDSC or mo-MDSC. However, mo-MDSC were the main subset in MDSC [87]. In another model, mo-MDSC, defined as CD11b^high^Ly6G^low^, exhibited a larger immunosuppressive activity than CD11b^high^Ly6G^high^G-MDSC counterpart [93].

In humans, data are still under investigation. Both mo- and G-MDSC subsets sorted from MM bone marrow are immunosuppressive, when cultured in ratio 1 : 1 with T cells stimulated by allogeneic dendritic cells [87]. Both mo- and G-MDSC subsets sorted from MM bone marrow or peripheral blood are immunosuppressive, cultured with autologous T cells for 4 days in the presence of T-cell stimulator factors, because G-MDSC are more immunosuppressive than mo-MDSC [88]. G-MDSC are increased in PB of MM patients and are able to induce the generation of Treg. G-CSF administered to induce stem cell mobilization caused an increase in the number of MDSC in the peripheral blood of patients with MM and a concentration of these immune-suppressive cells in peripheral blood stem cell collections [94, 95].

In our series, including 45 newly diagnosed MM and 60 MGUS, both G-MDSC and mo-MDSC were increased in PB of MM patients, while MGUS exhibited intermediate values between healthy subjects and MM patients. Myeloid compartment (identified as CD66 positive cells) and mature granulocytes exhibited immunosuppressive properties against allogeneic T cells stimulated with phytohemagglutinin (at increasing lymphoid : myeloid ratio) in both MGUS and MM patients, despite te fact that the effect was more evident in MM patients (Parrinello and Romano, manuscript in preparation). Granulocytes obtained from MM patients had greater amount of arginase-1 (a key mediator of G-MDSC immunosuppression) than MGUS or healthy subjects evaluated by RT-PCR (Parrinello and Romano, manuscript in preparation).

Emerging interest of MDSC in MM includes their involvement in other crucial processes for active disease, such as angiogenesis, since MDSC are able to release metalloproteinase-9 [96], and osteoclastogenesis, since MDSC can work as osteoclast progenitors [97, 98], thereby contributing to osteolytic bone disease in MM [99].

Several strategies are currently under investigation in human cancer to target MDSC in order to improve immune therapies:

1. deactivation of MDSC (using phosphodiesterase inhibitors, nitroaspirins, synthetic triterpenoids, COX2 inhibitors, ARG1 inhibitors, antiglycan antibodies, CSF-1R, IL-17 inhibitors, and histamine based approaches),
2. differentiation of MDSC into mature cells (with ATRA, vitamins A or D3, or IL-12),
3. inhibition of myeloid cell development into MDSC (with N-bisphosphonates, modulators of tyrosine kinases, and STAT3 inhibitors),
4. depletion of MDSC (using gemcitabine, HSP90 inhibitors, and paclitaxel) as recently reviewed [100].

Phosphodiesterase-5 (PDE-5) inhibitors, including sildenafil and tadalafil, inhibit the degradation of cyclic guanosine monophosphate (cGMP) leading to reduction in ARG1 and NOS2 expression, thus turning off the immunosuppressive property of MDSC [101]. In an in vitro model, sildenafil was able to restore expansion of T cells within the peripheral blood mononuclear cell fraction isolated from MM patients [101] thus leading to PDE5-inhibitors as novel immunotherapy in MM.

A phase II study has been presented at 2013 ASH annual meeting to test whether tadalafil could improve the response to lenalidomide and dexamethasone (NCT01374217 on http://www.clinicaltrials.gov/), in 13 patients who were refractory to lenalidomide-based regimens. However, the study was early stopped for lack of response and potentially of target, since MDSC were not detected in any of the patients at baseline in both blood and marrow and this correlated with the lack of clinical response [102]. However, the same group recently published the clinical benefit in an end-stage MM patient in whom responsiveness to lenalidomide-based therapy was restored upon the addition of tadalafil [103].

Novel agents active against MM, such as lenalidomide, and bortezomib inhibit NF-*κ*B activity as part of their diverse actions contributing to modulating proteins involved in antigen presentation [104]. Recently, modulation by lenalidomide of regulatory cells, with an immunophenotype overlapping with mo-MDSC, defined as CD33^+^CD11b^+^CD14^−^HLADR^−^, has been reported [89], while in vitro studies exclude a direct effect of lenalidomide or bortezomib in MDSC expansion [88]. On the other hand, in lymphoma-bearing mice, lenalidomide can reduce MDSC numbers and reverts cancer-induced immunosuppression [105].

Some chemotherapeutic agents used also in MM, such as cyclophosphamide or anthracyclines, have immunological side effects [106] that include MDSC expansion [107, 108] or inhibition [109], associated to T-cell function modulation, but this aspect has been poorly evaluated in MM.

Nitrogen-containing bisphosphonates, such as zoledronic acid, have a predominant role in the supportive therapy of MM patients [110] with benefit in survival [111–113]. Nitrogen-containing bisphosphonates inhibit bone resorbing osteoclasts, delay tumor growth rate as a consequence of MDSC depletion, and increased recruitment of T cells in murine models of solid tumors [99, 114]. However, no data are currently available about MDSC amount in MM patients treated with bisphosphonates.

## 5. Conclusions

MM microenvironment is a complex network, which progressively leads to functional impairment of host immune system. In the early stage of the disease, PC proliferation and migration are under the control of an active immune system, which fails to eradicate malignant cells. As long as the balance between immune effectors and PC is preserved, the disease remains completely indolent; this scenario seems to be consistent with the clinical course of MGUS patients. In a minority of subjects, several changes arise in terms of PC number and phenotype, expression of microenvironment-associated cytokines, and impairment in immune response and infiltration. MDSC increase, specific T-cell response is abolished, and finally PC become independent of microenvironment signaling, thus to confer an aggressive clinical course. Some drugs, including IMiDs, bisphosphonate, and cyclophosphamide, can interplay at this level, but use of drugs able of triggering this signaling is an urgent need to improve the efficacy of current treatments.
